# A Review of Three-Dimensional Electric Field Sensors

**DOI:** 10.3390/mi16070737

**Published:** 2025-06-24

**Authors:** Xiaonan Li, Yu Gu, Zehao Li, Zijian He, Pengfei Yang, Chunrong Peng

**Affiliations:** 1 School of Applied Science, Beijing Information Science and Technology University, Beijing 100192, China; 2023021095@bistu.edu.cn (X.L.); 2023021096@bistu.edu.cn (Y.G.); 2024021197@bistu.edu.cn (Z.L.); 2024021177@bistu.edu.cn (Z.H.); 2 Beijing Key Laboratory for Sensors, Beijing Information Science and Technology University, Beijing 100192, China; 3 State Key Laboratory of Transducer Technology, Aerospace Information Research Institute, Chinese Academy of Sciences, Beijing 100094, China; crpeng@mail.ie.ac.cn

**Keywords:** 3D EFSs, principle and design, decoupling calibration methods, sensitivity and resolution

## Abstract

Three-dimensional electric field sensors (3D EFSs) can simultaneously measure electric field components in three mutually orthogonal directions and comprehensively capture the spatial distribution and dynamic changes of the electric field. They can be widely used in atmospheric science, smart grids, aerospace, target detection, and other fields. This paper deeply analyzes the latest progress in 3D EFSs, focusing on four major types of sensors: DC field mill, electro-optic effect, capacitive sensing, and microelectromechanical system (MEMS). It elaborates on their working principles, structural design, and decoupling calibration methods. At the same time, the advantages and disadvantages of various types of 3D EFSs and their applications in different fields are analyzed. Finally, the challenges faced by 3D EFS technology and its future development direction are discussed.

## 1. Introduction

Electric field measurement is of irreplaceable importance for understanding complex physical phenomena, ensuring the safe operation of engineering systems, and promoting cutting-edge scientific research [1]. For example, in meteorology, atmospheric electric fields are monitored to track charge accumulation and transmission paths and to achieve lightning warning and climate change analysis [2,3]. In power systems, the electric field strength distribution around power equipment is monitored to evaluate the insulation status of equipment, prevent corona discharge and flashover failures, and provide reliable guarantees for electromagnetic environment assessment and safe operation of high-voltage transmission lines [4,5]. In industrial production, electric field measurements are used to solve problems such as static control, non-contact detection, and anti-interference testing [6].

As a vector field, the distribution and variation of the electric field in space usually need to be fully captured using multi-dimensional measurements. However, traditional electric field measurement techniques are often limited to a single dimension [7,8,9,10,11,12,13,14,15,16,17,18,19,20,21,22,23,24,25,26] or a two-dimensional plane [27,28,29], which greatly limits the complete characterization of electric field characteristics, especially in scenarios where the direction of the electric field is unknown or changes complexly. For example, in thunderstorms, charge separation inside the clouds will produce strong and complex electric field distributions [30]. In addition, the geometric structure and dielectric properties of buildings around high-voltage direct current transmission lines will have a significant distortion effect on the electric field distribution, making it difficult to determine the direction of the electric field [31]. Therefore, the emergence of 3D EFSs marks a major breakthrough in electric field measurement technology. They can simultaneously measure the components of the electric field in three orthogonal directions, thus making it possible to conduct a full-scale analysis of the electric field.

The presently described 3D EFSs can be classified into four categories: DC field mill, electro-optic effect, capacitive sensing, and MEMS sensors. DC field mill 3D EFSs are essentially categorized into two types: spherical EFSs and hybrid EFSs. Both are used for DC electric field measurements but have narrow bandwidths, large volumes, and are prone to mechanical wear. Additionally, their motor-driven operation results in relatively high power consumption. Of note, 3D EFSs based on the electro-optic effect employ the manipulation of the crystal refractive index by electric fields to modify the phase or intensity of light, facilitating non-contact monitoring of alternating or transient electric fields. Nevertheless, they are vulnerable to temperature variations, exhibit inadequate stability, necessitate intricate measurement systems, and incur high costs. Power consumption is primarily focused on the light source and electronics, with total consumption at the milliwatt (mW) level, which is comparatively minimal. Capacitive 3D EFSs are mostly utilized for AC electric field measurements; nevertheless, they exhibit restricted measurement ranges and diminished susceptibility to interference. MEMS 3D EFSs, characterized by their compact size, minimal power consumption (μW-mW range), and scalability for extensive deployment, have emerged as a pivotal area of advancement and investigation in three-dimensional electric field measurement.

This paper reviews the latest research progress of 3D EFSs, covering their working principles, structural design, decoupling calibration methods, and application cases in different fields. Through a systematic analysis of various types of 3D EFSs (including DC field mill type, electro-optic effect type, capacitive sensing type, and MEMS type, etc.), the challenges and future development trends of 3D EFSs are pointed out.

## 2. Field Mill 3D DC EFSs

### 2.1. Working Principle

**Figure 1:**
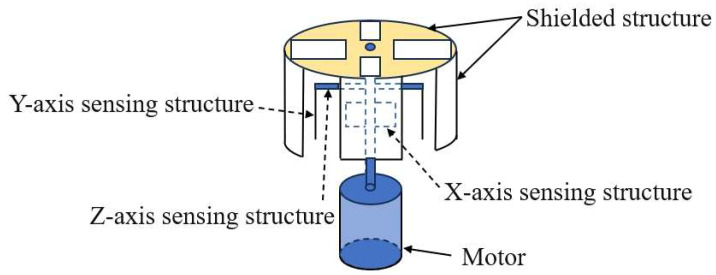
A simple diagram of a field mill 3D EFS.

The field mill 3D EFS is a classic electric field measurement device, primarily used to measure the DC electric field based on the charge induction principle. This type of sensor mainly consists of key components such as the shielding structure, induction structure, photoelectric encoder, and motor. During the operation of the equipment, the continuous rotation of the shielding structure (including the ball cage structure, electrode structure, spherical structure, and shielding cover structure) causes the area of the induction structure (including the spherical structure, electrode structure, and circular induction plate structure) exposed to the electric field to show regular periodic changes. Correspondingly, the induced charge on the induction structure will also change, thereby generating an induced current. With the help of the signal processing unit to accurately analyze and process the induced current, effective measurement of the DC electric field can be achieved. The basic structure of a field mill 3D EFS is shown in Figure 1.

According to Gauss’s theorem, the total induced charge within the closed surface *A* is expressed as follows:

(1) $$\sum_{A}q\left(t\right)=\varepsilon \Psi =\iint_{A}\varepsilon \vec{E}\cdot d\vec{A}\left(t\right)$$

Here, ε is the dielectric constant, Ψ is the electric flux, and $\vec{E}$ is the component of the electric field strength perpendicular to the surface of the sensing electrode.

The induced current $\mathrm{i}\left(t\right)$ on each inductive structure can be calculated as follows:

(2) $$\mathrm{i}\left(t\right)=\frac{d\Sigma_{A}q\left(t\right)}{dt}=\frac{d\iint_{A}\varepsilon \vec{E}\cdot d\vec{A}\left(t\right)}{dt}$$

The induced current is processed by the subsequent circuit to obtain the output voltage, thereby enabling the measurement of the DC electric field.

### 2.2. Prototype Instantiation for Field Mill 3D DC EFSs

The field mill DC 3D EFS has shown a wide range of potential applications in fields such as meteorology, power grids, and underwater electric field detection. The following discusses the exploration and research in these areas.

**Figure 2:**
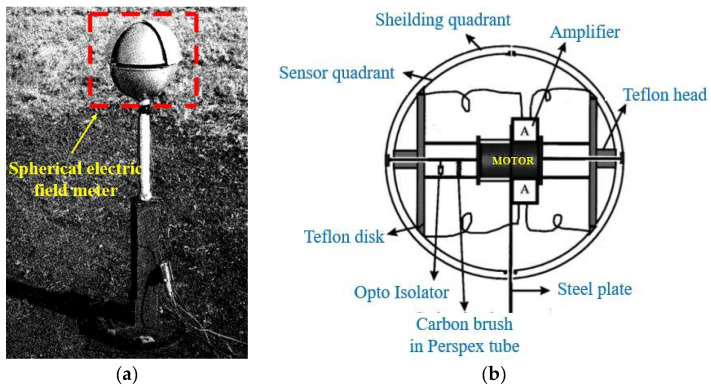
Two types of field mill 3D EFS structures with (**a**,**b**) corresponding to [32,33], respectively.

In the field of meteorology, in 1983, Kamra reported a spherical three-dimensional electric field meter (3D EFM) [32] that can be used to measure the 3D electric field vector of the atmosphere, as shown in Figure 2a. The device fixes the aluminum quadrant on a steel base and strip to form a spherical cage structure, which contains a synchronous AC motor. This allows the sensor quadrant to rotate, alternately exposed to and shielded from the external electric field. In the electric field range of 0–50 V/m, the sensitivity is 2 V/m. This design solves the problem that traditional EFMs can only measure the vertical component of the electric field. However, there is inherent noise due to the on-off contact problem between the carbon brush and the commutator, which affects the measurement accuracy. In 1999, Ravichandran and his team made technical improvements based on the 1983 design [33]. As shown in Figure 2b, a static quadrant sensor design was adopted. Through subsequent electronic rectification and signal amplification, the noise interference caused by mechanical contact was effectively eliminated, thereby improving measurement accuracy. $E_{z}$ is usually between tens of volts/meter and hundreds of volts/meter in clear weather, and the electric field of the horizontal component may reach 5% of the vertical component. The amplitude of the sensor’s output signal fluctuation is less than the electric field strength of ±20 mV, indicating a low noise level.

**Figure 3:**
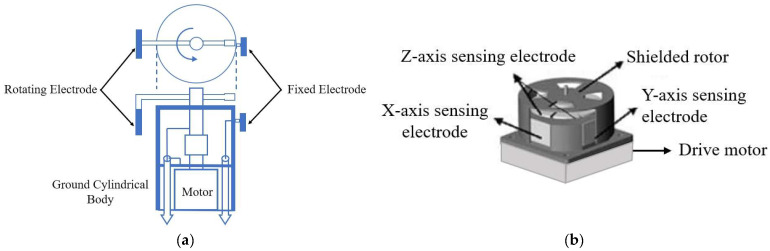
Two types of field mill 3D EFS structures with (**a**,**b**) corresponding to [34,35], respectively. The blue arrow represents the signal transmission, which is subsequently connected to the AD converter. The dotted line represents the extension of the top view of the rotating structure.

In 2006, Tantisattayakul et al. developed an innovative EFS that can concurrently measure the vertical and horizontal electric fields of thunderclouds [34]. Figure 3a illustrates that this device employs a grounded cylinder, a spinning electrode, and a stationary electrode to assess the electric field. Experimental assessments demonstrate a strong linear association between the charge on the fixed electrode and $E_{z}$, whereas the measured values for the horizontal field component $E_{r}$ align closely with those obtained from boundary element method simulations. The revolving electrode (26 revolutions per second) and data capture (2 kS/s) offer millisecond-level temporal resolution. The apex orientation of the horizontal field component can be ascertained throughout the rotating phase. This EFS fulfills the requirement for electric field measurements under thundercloud atmospheric circumstances, offering more extensive data for analyzing the charge distribution of thunderclouds. Nonetheless, it fails to consider the impact of the ground’s dielectric constant on the electric field. In 2008, Zheng et al. reported a new structure for a 3D EFS based on the charge induction principle [35], as shown in Figure 3b. Within the electric field measurement range of ±30 kV/m, the resolution can reach 20 V/m, the sensitivity can reach 50 V/m, and the linearity is better than 1%. Their team conducted a series of in-depth research and discussions surrounding this structure.

**Figure 4:**
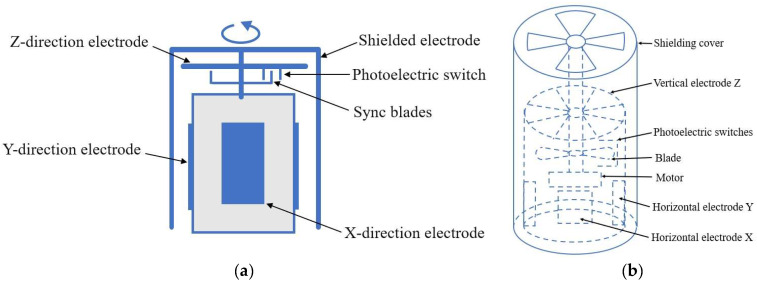
Two types of field mill 3D EFS structures with (**a**,**b**) corresponding to [36,37], respectively. The blue arrow represents the rotation direction of the sensor shield structure. The dotted line represents the internal structure of the sensor.

In 2018, Xing et al. introduced a 3D atmospheric electric field measurement device that combines a 3D electric field measurement unit and a dielectric constant measurement unit [36], as shown in Figure 4a. The three electric field components along the *X*, *Y*, and *Z* axes are measured by three sets of orthogonal electrostatic induction units. Observation results on sunny days show that the average values of $E_{x}$, $E_{y}$, and $E_{z}$ are 0.0682 (kV/m), −0.049 (kV/m), and 0.6513 (kV/m), respectively. In thunderstorm conditions, the peak value of $E_{z}$ can reach several kV/m, which can detect rapid electric field changes caused by lightning discharge activities and is consistent with radar echo results with high accuracy. This approach solves the problem of neglecting the influence of the ground dielectric constant on electric field measurement in previous studies, thereby improving the accuracy and reliability of the measurement results. In 2024, Lou proposed a field-milling 3D atmospheric EFS [37]. As shown in Figure 4b, based on the characteristic of metal conductors generating induced charges in the electric field, the 3D electric field strength is measured. The minimum electric field resolution measured in a thunderstorm cloud environment is 10 V/m, and the average values of $E_{x}$, $E_{y}$, and $E_{z}$ are approximately 60 (kV/m), 10 (kV/m), and 55 (kV/m), respectively. This effectively improves the accuracy of thunderstorm cloud warnings.

**Figure 5:**
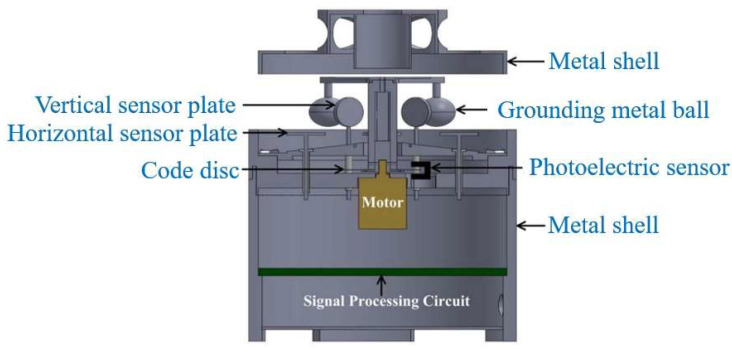
A novel sensor for the measurement of the 3D component of the synthetic electric field vector in a high-voltage direct current transmission line.

In the field of power grids, Liu et al. innovatively proposed a new type of EFS with a 3D field mill structure in 2023 [38]. As shown in Figure 5, this device uses a signal processing circuit to accurately read 3D electric field data. The measurement curves of the linear relationship between the output and input electric fields were obtained in the three directions of x, y, and z, with $R^{2}>0.984$ and RMSE < 0.0153. In the random angle measurement experiment, the maximum relative error between the predicted electric field amplitude and the actual electric field amplitude was 6.79%. This sensor can be widely used in electromagnetic environment monitoring around high-voltage DC transmission lines.

**Figure 6:**
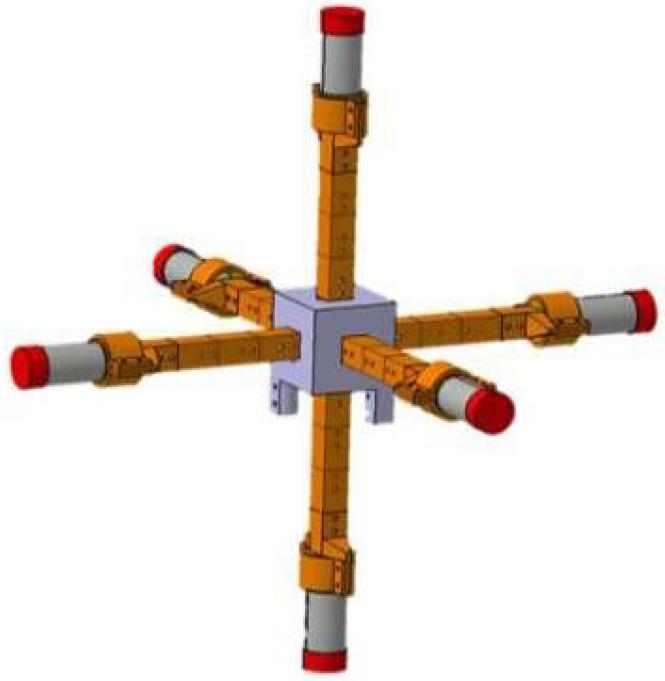
A 3D EFS applied underwater.

In the field of underwater electric field detection, in 2011, Kim et al. introduced a three-axis underwater potential sensor utilizing an insulated cube (5 cm × 5 cm × 5 cm) equipped with six metal electrodes on its sides [39]. The electric field strength was estimated by measuring the potential difference in orthogonal directions, a method initially utilized to identify leakage currents in submerged manholes. The sensor can identify horizontal leakage sources by employing the polarity reversal characteristic of potential differences. Experimental findings demonstrate that positioning the sensor at the base of the manhole produces optimal performance. Experiments validated that the sensor could identify potential variations of ≥100 mV in a 220 V/60 Hz leaking context. Nonetheless, the sensor’s fundamental performance metrics, including sensitivity and resolution, were not measured, and essential data regarding environmental adaptability—such as temperature and humidity, electromagnetic interference (EMI)/electromagnetic compatibility (EMC), pressure dependency, and power consumption—were absent. Additional research is required to improve the engineering feasibility of the sensor. In 2022 and 2024, Jason Kim et al. proposed a three-axis underwater EFS that achieves underwater 3D electric field measurement through a six-electrode configuration [40,41]. As shown in Figure 6, to address the problem of inaccurate measurement caused by the axial error of the sensor, a mechanical system was constructed to calibrate the sensor. The axial error calibration accuracy can reach within 0.03°, the deviation between the electric field measurement and the theoretical value is 1.2 μV/m, and the average positioning error is 2.85 cm, which improves both electric field measurement accuracy and underwater positioning accuracy. This demonstrates that the system has significant practical application value for tasks requiring accurate 3D positioning of underwater objects, such as underwater detection, construction, and monitoring.

## 3. 3D Optical EFSs

Three-dimensional optical EFSs have received extensive attention and application due to their small size, wide frequency response range, high sensitivity, high resolution, strong resistance to electromagnetic interference, inherent insulation characteristics, high electrical safety, and suitability for long-distance measurement and signal transmission. Currently, this type of EFS is used to evaluate the electromagnetic compatibility of power equipment, measure the electromagnetic field distribution around the equipment, and help improve the design and performance of the equipment. It is also applied to study cloud charge distribution and lightning mechanisms, which helps improve the accuracy of weather forecasts.

### 3.1. Working Principle

**Figure 7:**
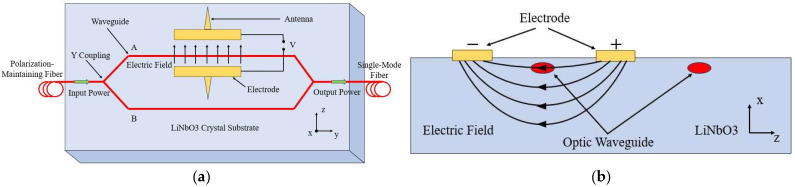
Typical structure of a MZI sensor: (**a**) The basic structure of a MZI; (**b**) Cross section view.

Three-dimensional optical EFSs utilize the linear electro-optic effect (Pockels effect) to alter the phase or intensity of light by affecting the refractive index of the crystal through the electric field, thereby enabling non-contact measurement of the electric field. Most of these sensors are based on three integrated optical waveguide Mach–Zehnder interferometer (MZI) modulators to measure the electric field E (comprising $E_{x}$, $E_{y}$, and $E_{z}$) in three directions. One of the typical structures of the MZI sensor is shown in Figure 7 [42,43].

The input light beam is first evenly split into two parts by the *Y*-type coupler on the left and then propagates along two horizontal waveguides (waveguide *A* and waveguide *B*), respectively. In waveguide *A*, the light beam is modulated by the electric field applied by the electrodes and antenna. The two beams interfere after passing through the *Y*-type coupler on the right. When the electric field acts on the dipole antenna, an induced voltage is generated at both ends of the electrodes of the modulator. As a result, the output power of the sensor corresponds to an optical signal that is proportional to the applied electric field strength. Most 3D optical EFSs use LiNbO_3_ electro-optical crystals, where the refractive index changes under the influence of the electric field. The phase shift calculation formula for the light wave during propagation is as follows:

(3) $$\varphi =\frac{\pi n^{3}rL\Gamma }{\lambda }E$$

where *n* is the intrinsic refractive index of the crystal, *r* is the electro-optic coefficient corresponding to the polarization of the electric field $\vec{E}$, *L* is the length of the electrode, λ is the wavelength, and Γ(<1) is the overlap factor between the electric field and the light wave in the crystal.

According to the basic principle of the Mach–Zehnder modulator, the optical power output by the modulator can be expressed as follows:

(4) $$P_{0}=\frac{P_{i}}{2}\left[1+\cos\left(\Delta \varphi_{0}+\varphi \right)\right]$$

where $P_{0}$ and $P_{i}$ are the output and input laser powers, respectively, and $\Delta \varphi_{0}$ is the intrinsic phase difference between the two waveguides. When $\Delta \varphi_{0}=\frac{\pi }{2}$, the output optical power is expressed as follows:

(5) $$P_{0}=\frac{P_{i}}{2}\left[1-\sin\varphi \right]$$

If φ≪1, the above formula can be simplified to the following:

(6) $$P_{0}=\frac{P_{i}}{2}\left[1-\varphi \right]$$

Substituting Equation (3) into the calculation, we obtain the following:

(7) $$P_{0}=\frac{P_{i}}{2}-\frac{\pi n^{3}rL\Gamma P_{i}}{2\lambda }E$$

Obviously, when the sensor operates near the static operating point, the output laser power is linearly related to the measured electric field strength. The electric field value can be determined by measuring the output laser power.

### 3.2. Prototype Instantiation for 3D Optical EFSs

Three-dimensional optical EFSs typically include multiple mutually perpendicular electric field detection units to ensure accurate capture of the spatial distribution of the electric field. These sensors are primarily used to measure alternating or transient electric field signals, such as lightning electromagnetic pulses or transient voltages in power systems. They have broad application prospects and can be used for power equipment monitoring, electromagnetic compatibility testing, and lightning protection.

**Figure 8:**
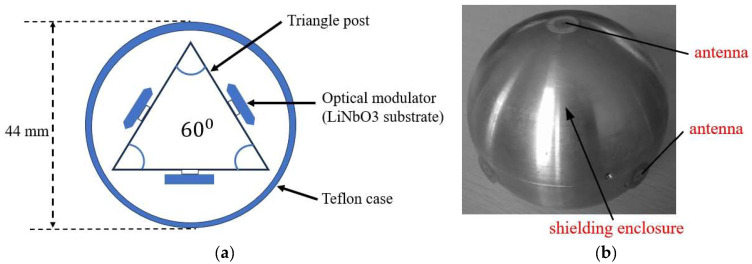

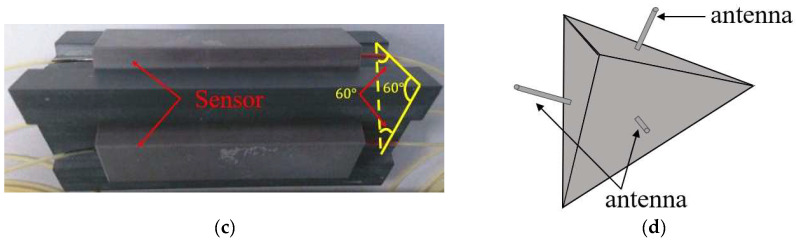
Four types of optical 3D EFSs with (**a**–**d**) corresponding to [44,46,47,48], respectively.

In 2002, Tajima et al. created an optical isotropic 3D EFS employing a MZI [44]. Figure 8a illustrates that this sensor attains a bandwidth of 10 GHz through the integration of electrode downsizing (1 mm) and adjustment for transit time effects using a three-axis orthogonal optical interferometer probe. The minimum observable electric field strength in GTEM cells and semi-anechoic chambers was 22 mV/m, with a directional sensitivity deviation of ±1 dB. The sensitivity at 5 GHz was ±3 dB, while at 10 GHz it was ±5 dB. As the temperature fluctuated between 0 °C and 40 °C, the sensitivity deviation maintained within 3 dB, exhibiting no substantial drift. In 2010, Garzarella et al. introduced a quasi-longitudinal configuration electro-optic crystal-based all-dielectric 3D EFS [45]. The quasi-longitudinal optical path design improves stability, diminishes phase noise, and eradicates crystal-induced phase drift, thereby resolving calibration instability problems arising from fiber vibrations or temperature fluctuations in conventional sensors. The all-dielectric passive design mitigates interference from conventional dipole antennas on the measured electric field, and the sensor obviates the necessity for compensatory optical components. Experimental measurements demonstrated a sensitivity of 0.25 mV/m-$Hz^{1/2}$ and a bandwidth of 14 GHz, exhibiting linear performance under applied electric fields from 1 V/m to 100 V/m within the frequency range of 1 MHz to 10 GHz. It functions reliably in intense electromagnetic fields, exhibiting undistorted waveforms and superior EMI/EMC performance. Nonetheless, the verification of environmental adaptability is inadequate, and subsequent studies should incorporate temperature and humidity assessments. Furthermore, inter-axis interference remains unverified, potentially compromising measurement accuracy in intricate domains. In 2012, Si et al. proposed a spherical fiber-optic transmission 3D EFS [46], which measures the 3D electric field in electromagnetic pulses based on the electro-optic effect. As shown in Figure 8b, the sensor adopts an embedded spherical structure, which not only effectively reduces interference to the measured electric field but also serves as a shielding cover, improving the sensor’s shielding efficiency to 60 dB. This sensor causes minimal disturbance to the measured electric field, with the inter-electrode coupling coefficient being less than 6%, and the 3D field angle and peak synthesis error being less than 5%. However, the frequency response range is not specified, and the ability to measure electric fields at higher frequencies may be limited. In 2014, Zhang et al. developed a 3D integrated optical EFS [47]. Figure 8c illustrates that this sensor employs three optical waveguide MZIs for electric field detection. The working mechanism entails modulating the laser beam with an induced voltage and employing the electro-optic effect of LiNbO_3_ crystals to obtain electric field information. The sensitivities along the *X*, *Y*, and *Z* axes are 1.1 mV/kV/m, 1.7 mV/kV/m, and 1.4 mV/kV/m, respectively. The linear fitting correlation coefficients for all three axes across the electric field range of 15–370 kV/m are approximately 0.999, indicating exceptional linearity. The sensor can identify electric fields above 1000 kV/m and possesses a frequency response of up to 500 MHz, rendering it appropriate for measuring lightning electromagnetic pulses. In 2017, Yan et al. introduced a 3D pulse electric field test system [48]. As shown in Figure 8d, three mutually perpendicular triangular cone antennas are used to receive 3D transient pulse electric field signals. The electric field signals are then converted into laser signals through signal synthesis and electro-optical modulation, followed by measurement via optical fiber transmission. The test system exhibits good broadband characteristics and can measure 3D pulse electric fields. Its bandwidth ranges from 1 kHz to 1 GHz, and its dynamic range reaches 60 dB.

**Figure 9:**
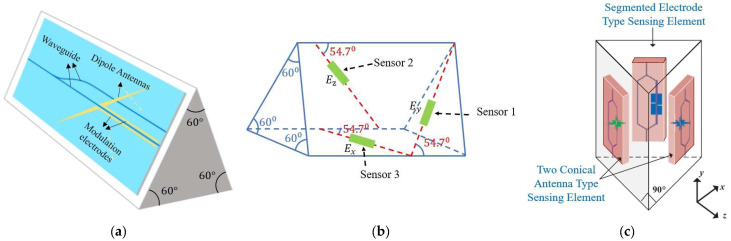
Three types of optical 3D EFSs: (**a**) corresponding to [49], (**b**) adapted from [51], and (**c**) corresponding to [52]. Green represents one-dimensional electric field sensors, blue represents one-dimensional electric field sensors, and yellow represents Modulation electrodes.

In 2023, Liu et al. proposed a 3D broadband EFS based on an integrated lithium niobate insulator platform [49], as shown in Figure 9a. The sensor achieves 3D electric field measurements by encapsulating three sensor chips in a triangular prism-shaped fixture. The optical waveguide on each chip uses an asymmetric Michelson interferometer architecture. A tilted dipole antenna is positioned at a certain angle to the optical waveguide. The three pairs of antennas are orthogonal to each other, allowing the sensor to measure the electric field in three orthogonal polarization directions. The frequency response range of the sensor spans from 10 MHz to 3 GHz, and the relative measurement error of the electric field amplitude in each polarization direction is less than 5.1%. In 2023, Zhu et al. introduced a 3D EFS utilizing optically suspended charged nanoparticles. Nanoparticles are confined in a vacuum via a laser, and their mechanical reaction to electric fields is employed for three-dimensional electric field measurements [50]. At $1.4\times 10^{-7}$ mbar, the sensitivity for force detection of a 100*e* charge attained $10^{-20}N∕{Hz}^{1/2}$, whereas the sensitivity for electric field strength detection achieved $1\;\mu V\cdot {cm}^{-1}\cdot {Hz}^{-1/2}$. The spatial resolution can attain the nanoscale scale, with a linear measuring range of 91 dB and a maximum detectable electric field of 6.5 MV/m. The noise-equivalent electric field strength bandwidths are 48.6 kHz at 10^−4^ mbar and 1.1 kHz in high vacuum, respectively, within 3 dB of the thermodynamic limit. Feedback cooling lowers particle temperature to 5.1 mK, mitigating thermal noise and ensuring temperature stability. This EFS, while exhibiting ultra-high sensitivity, necessitates vacuum conditions, hence augmenting system complexity. In 2024, Zhang et al. developed a fully dielectric 3D EFS architecture with a regular triangular prism [51], as illustrated in Figure 9b. Each sensor has dimensions of 25 mm × 25 mm × 90 mm, and a 54.7° installation angle is employed to ensure natural orthogonality among the three sensors. The entirely dielectric configuration of the sensors mitigates electric field distortion and diminishes temperature drift. Resin encapsulation and fiber optic transmission were utilized, offering substantial resistance to EMI. The sensor’s linear measurement ranges along the *X*-, *Y*-, and *Z*-axes are 3.71 kV/m to 388 kV/m, 2.78 kV/m to 403 kV/m, and 4.50 kV/m to 375 kV/m, respectively, having sensitivities of 0.908 mV/kV/m, 1.043 mV/kV/m, and 0.781 mV/kV/m for the *X*-, *Y*-, and *Z*-axes, respectively. The linear correlation values for all three axes surpass 0.999, indicating exceptional linearity. The sensor rotates 360° in the YOZ and XOY planes, exhibiting a measurement error of less than 4.97%. This construction has not been subjected to three-axis calibration testing. In 2024, Long et al. designed a 3D integrated optical waveguide EFS [52] that uses an asymmetric waveguide structure and segmented electrodes, as shown in Figure 9c. The sensor can accurately capture the temporal waveform of the lightning pulse electric field, offers good directivity, and has a frequency response range from 0 GHz to 7 GHz. The maximum relative error is less than 3% in the range of 8–60 kV/m for pulse electric fields, and it can measure the electric field strength accurately in any direction in space. In 2025, their team implemented technical enhancements to the original idea and built a three-dimensional electric field monitoring device [53]. The sensor volume was lowered by 92% relative to conventional electromagnetic probes. An automatic working point tracking system was implemented to dynamically adjust for temperature drift and variations in light sources. The packaging employed a dielectric shell to prevent electromagnetic interference from metal components. Experiments on lightning electromagnetic fields were performed following the IEC 61000-4-5 testing standard [54]. The electric field measurement range spans from 5 kV/m to 50 kV/m, with a measurement bandwidth encompassing power frequency to lightning transients (50 Hz to 100 kHz). The sensitivity is 0.0291 mV/(kV/m), and the linear correlation coefficient remains at 0.9994. The accuracy of power frequency measurement surpasses 97%, while the relative error of the synthesized three-dimensional electric field measurement is less than 4%. The system employs a tunable laser source with a power consumption of 3 mW. At 26.6 °C, the sensor delivers an output power of 11.246 mW, providing a low-power benefit.

## 4. 3D Capacitive EFSs

### 4.1. Working Principle

The core principle of a 3D capacitive EFS is to capture the spatial electric field components through multi-axis, orthogonally distributed sensing electrodes and reconstruct the 3D electric field vector by applying signal processing and calibration algorithms. The electrode structure may include a spherical design, parallel plate, or PCB copper foil. The electric field induces a charge on the electrode surface, which is converted into a voltage signal by measuring the capacitance change or through charge integration. This signal is then converted into a measurable electrical signal via optics (fiber optic transmission) or a circuit system (differential integration circuit), allowing the electric field information to be measured.

### 4.2. Prototype Instantiation for 3D Capacitive EFSs

**Figure 10:**
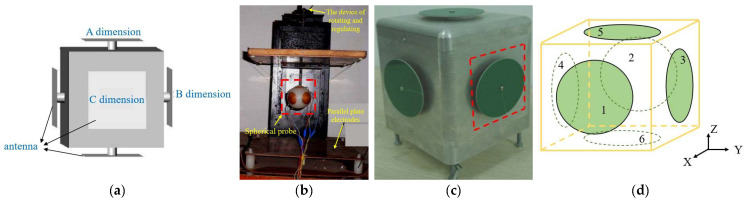
Four types of 3D capacitive EFSs with (**a**–**d**) corresponding to [56,57,58,60], respectively.

In 1983, Misakian et al. systematically studied the measurement performance of a free-body EFM in a non-uniform electric field for the first time [55]. The study used the induced charge of the EFM to measure the electric field strength. The study concluded that as long as the electrodes of the EFM are hemispherical or symmetrically rectangular, the EFM calibrated with a uniform electric field can be used to measure non-uniform power-frequency electric fields with minimal error. However, the study mainly focused on theoretical analysis and experimental verification under ideal conditions and did not conduct in-depth research on high-frequency electric field measurements in actual complex environments. In 2007, Xu et al. proposed a 3D parallel plate EFS for transient electric field measurement [56]. As shown in Figure 10a, the electric field signal is received by a 3D antenna, converted into an induced voltage, and then measured using an optical fiber transmission system, thus avoiding the loss of phase information and measurement errors that arise from multiple measurements in traditional methods. The sensor can measure electric field strengths up to 16,032 V/m, with a peak error of 2.2% and a bandwidth of 730 MHz. It demonstrates good linear response and high accuracy, effectively addressing the measurement challenges of high-frequency transient electric fields. In 2014, Zhang et al. proposed a spherical EFS probe structure consisting of six mutually insulated symmetrical electrodes [57]. As shown in Figure 10b, the sensor system exhibits a nonlinear error of about 6.68%, a sensitivity of 3.03 mV/kV/m, a resolution of 2.5%, and good linearity when the industrial frequency electric field strength is less than 12 kV/m. In 2015, Jiang et al. proposed an integrated 3D EFS [58] for simultaneously measuring the horizontal and vertical electric fields generated by lightning discharge. As shown in Figure 10c, this sensor detects the electric field through orthogonal electrostatic antennas. The sensor exhibits a flat response in the frequency range from 1 kHz to 10 MHz, and the interference between the vertical and horizontal electric fields is less than 1.2%. Two differential integrators were engineered to quantify the electric field, attaining a common-mode rejection ratio (CMRR) over 60 dB, therefore efficiently mitigating common-mode noise. In 2016, Rumyantseva et al. proposed a new method for measuring electric field strength using a 3D inductive spherical sensor [59]. The method employs the sensor to measure electric field strength at a specific position in space. By adjusting the direction and rotation of the sensor to balance the components of the electric field strength vector, a measurement with an error of less than 2% can be achieved over a larger spatial range. In 2021, Suo et al. introduced a hexahedral three-dimensional structure for measuring power frequency electric fields with a parallel plate probe [60], as seen in Figure 10d. An equipotential ring was engineered to mitigate electric field distortion at the peripheries of the parallel plates, thus resolving the nonlinear error complications induced by edge effects in conventional probes. The hexahedron is constructed using ABS material, which possesses superior electrical insulating qualities and remains mostly impervious to temperature, humidity, or frequency variations. The system’s measurement range spans from 1 kV/m to 200 kV/m, featuring a resolution of ≥1 V/m, a nonlinear error of 2.15%, an uncertainty of ±0.55%, a sensitivity of 19.10 mV/($kV\cdot m^{-1}$), and a capacitance temperature drift coefficient of 33 ppm/°C. The impact of a 2 °C temperature variation is insignificant.

**Figure 11:**
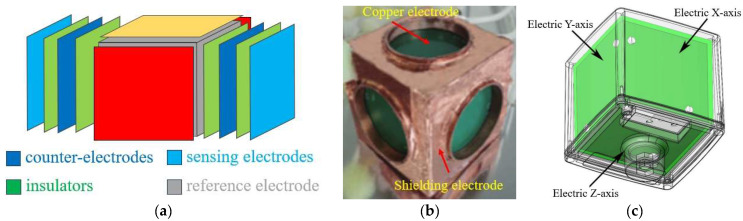
Three types of 3D capacitive EFSs with (**a**,**b**) corresponding to [61,62], respectively, and the EHP-50F product diagram (**c**).

In 2023, Roblin et al. created a system that integrates a three-axis electrode capacitive EFS with a spherical point charge source, which is upheld by a conical structure [61], as seen in Figure 11a. The three-axis sensor consists of detecting electrodes, a counter-electrode, and a reference electrode and was experimentally confirmed using two-dimensional scanning and a mirror charge model. In a double-sphere monopole arrangement, the sensor recorded the *X*-component of the electric field with a comparative error of 2.1% compared to the model, and the relative error was within ±5% in 85% of the area. The system’s dynamic range encompasses the near-field region at 110 V/m. The sensor functions within the frequency range of 100 Hz to 10 kHz; however, parameters including temperature, humidity, electromagnetic compatibility, pressure dependency, and power consumption were not disclosed. In 2024, Zhao et al. proposed a 3D EFS with low inter-axis coupling [62]. As shown in Figure 11b, a 3D EFS capacitive sensing unit with a shielding electrode was developed, with PCB copper cladding used as the detection unit. The measurement deviation of the 3D EFS with the shielding electrode is within 3.2%, which is 12% lower than the deviation of the 3D EFS without the shielding electrode. The sensor can measure electric field strengths ranging from at least 1.25 kV/m to 2.5 kV/m. The 3D EFS based on the electric field shielding structure can decouple more reliably and effectively reduce the measurement deviation of the spatial electric field.

The EHP-50F low-frequency electric field isotropic probe analyzer, produced by the German company Narda, is depicted in Figure 11c. It comprises three orthogonal parallel-plate capacitors, engineered for electric field measurements within the frequency spectrum of 1 Hz to 400 kHz. This instrument offers concurrent measurement over the *X*, *Y*, and *Z* axes and has a robust integrated spectrum analyzer. The electric field measurement range is 5 mV/m to 1 kV/m (low range) and 500 mV/m to 100 kV/m (high range), with a resolution of 0.1 mV/m (low range) and 1 mV/m (high range). The attenuation of the electric field by the magnetic field is <10 V/m at 1 mT, in accordance with the EN 61326-1 (2013) standard for electromagnetic compatibility in industrial settings [63]. It has successfully undergone radiation emission and immunity testing, exhibiting robust interference resistance. The expanded uncertainty for the electric field is ≤8.1% at 50 Hz (industrial frequency) and ≤10.3% across the broadband range of 5 Hz to 100 kHz, conforming to the IEC 61786 standard [64] for field instruments. The protection rating is IP42, and the operational temperature range is −20 °C to +55 °C, with a temperature coefficient of $-4\times 10^{-3\;}dB/℃$ (referred to 23 °C). At 23 °C, the standard relative humidity deviation is $+11\times 10^{-3}dB/\%$ (10–50% RH) and $+22\times 10^{-3}dB/\%$ (50–90% RH), rendering it appropriate for diverse intricate measurement settings, including high-voltage transformers and power lines.

It is worth noting that the interface circuit design of capacitive sensors is crucial to frequency responsiveness. The interface design suggested by Pullano et al., utilizing the second-generation voltage conveyor [65], attains a transimpedance gain of 86 dBΩ and a bandwidth of 103 kHz without filtering, while consuming merely 6 mA of power. This low-power design with a wide frequency response offers a technical solution for implementing 3D capacitive EFSs in resource-limited systems.

## 5. MEMS 3D EFSs

### 5.1. Working Principle and Classification

Three-dimensional electric field microsensors based on MEMSs have become a research hotspot due to their small size, low power consumption, and suitability for large-scale sensor arrays. Over the past two decades, teams primarily led by the Chinese Academy of Sciences have developed several typical 3D EFS structures based on the charge induction principle to achieve high-precision electric field measurements. The charge induction principle is similar to the working principle of the field-grinding 3D EFS. It requires a driving structure to periodically move the shielding electrode, thereby periodically shielding the electric field on the sensing electrode, which continuously generates an induced current. This method solves the problem that the electrostatic field and low-frequency electric field cannot continuously provide energy. The main types of MEMS 3D EFSs are single-chip structures and assembled structures. The single-chip structure is mainly based on coplanar electrodes, while the assembled structure includes planar, triangular prism, and cubic structures.

### 5.2. Prototype Instantiation for MEMS 3D EFSs

#### 5.2.1. Single-Chip 3D MEMS EFSs

**Figure 12:**
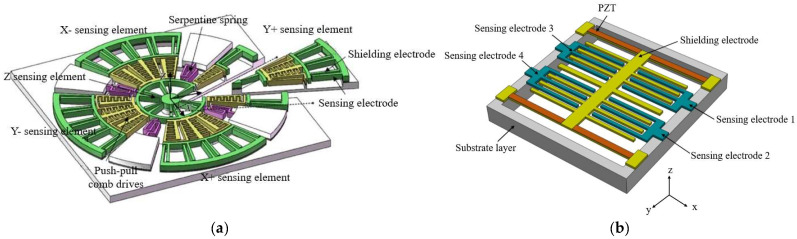
Two MEMS 3D EFSs in a single-chip structure with (**a**,**b**) corresponding to [66,68], respectively.

The single-chip 3D MEMS EFS integrates a three-axis electric field-sensitive structure onto a single chip to achieve the measurement of 3D electric fields. In 2017, Ling et al. first reported a single-chip 3D electric field microsensor [66,67], as shown in Figure 12a, which overcomes the limitations of traditional multi-chip assembly. The *Z*-axis component of the electric field is detected by placing a sensing element at the center of the chip, while the *X*-axis and *Y*-axis components are measured using two pairs of sensing elements in a cross configuration. A plane rotation mechanism enables simultaneous detection. In the range of 0 to 50 kV/m, the linear error of the sensor is within 5.5%, and the *X*-, *Y*-, and *Z*-axis sensitivities are 0.136 $mV\cdot kV^{-1}\cdot m$, 0.121 $mV\cdot kV^{-1}\cdot m$, and 0.101 $mV\cdot kV^{-1}\cdot m$, respectively, with a total measurement error of less than 14.04%. This design effectively solves the problems of large volume and low integration associated with traditional multi-chip assembly solutions. In 2023, Peng et al. proposed a single-chip 3D electric field microsensor based on piezoelectric drive [68]. As shown in Figure 12b, a set of shielding electrodes and four sets of symmetrically distributed sensing electrode structures are used to measure the 3D electric field. Piezoelectric excitation is employed to reduce the excitation voltage and cross-interference noise, thereby improving the performance of the microsensor. Under a 2V AC driving voltage, the device’s sensitivities on the *X*-, *Y*-, and *Z*-axes are 0.2315 mV/kV/m, 0.3727 mV/kV/m, and 2.187 mV/kV/m, respectively. The linear errors along the three coordinate axes are all within 2.77% in the range of 0 to 50 kV/m, and the maximum measurement error of the 3D EFM is less than 8%.

#### 5.2.2. Assembled 3D MEMS EFSs

Although the single-chip 3D MEMS EFS has a compact structure and high integration, it may not achieve sufficient sensitivity or dynamic range due to space limitations. In contrast, the sensitive structures of each axis in the assembled structure are relatively independent, which helps to better address the problem of inter-axis coupling. The assembled 3D MEMS EFS consists of three one-dimensional electric field-sensitive chips that form three orthogonal sensing axes.

**Figure 13:**
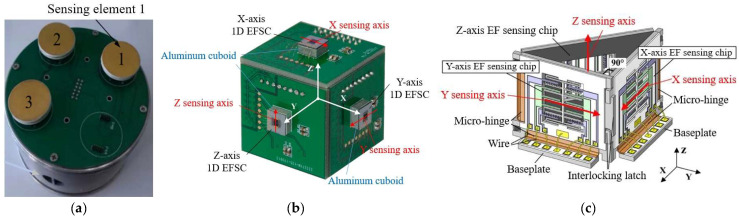
Three MEMS 3D EFSs in an assembled structure with (**a**–**c**) corresponding to [69,70,71], respectively.

In 2014, Wen et al. proposed a 3D electric field measurement method based on a coplanar decoupling structure [69]. As shown in Figure 13a, by creating and deriving a coupling sensitivity matrix, three electric field sensing elements distributed on a plane can be used to measure the 3D electric field, effectively eliminating cross-interference between these sensing elements. Through finite element simulation, the error is less than 2.5%. Experimental results show that the average error of the developed probe prototype in measuring the electric field is 4.4%. In 2018, Ling et al. proposed a new cubic 3D electric field microsensor [70]. As shown in Figure 13b, this device is designed by creating each one-dimensional EFS chip as a symmetrical structure, forming a pair of sensing structures symmetrically placed in the plane. Through a differential circuit, it can sense the electric field parallel to the symmetrical structure and eliminate cross-axis coupling interference. Silicon-on-insulator (SOI) wafers are used, and micromachining technologies, such as deep reactive ion etching, are employed to form sensing electrodes, shielding electrodes, push-pull comb actuators, and folded beams. In the electric field range of 0–120 kV/m, the cross-axis sensitivity is better than 5.48%, and the total measurement error is within 6.16%. In 2019, their team introduced a new chip assembly form [71]. As shown in Figure 13c, the EFS takes the shape of a triangular prism, with three electric field-sensitive chips placed on the three sides. Photosensitive polyimide is introduced into the micromachining process of SOI to manufacture flexible micro-hinges, turning independent components into foldable integrated structures, and ensuring the orthogonality of the three sensing axes through interlocking locks. The volume after microassembly is only about 364.5 mm^3^. The cross-axis sensitivity of the 3D EFS is within 19.54%, the linearity error is less than 4.30%, and the 3D electric field measurement error is within 9.33%.

**Figure 14:**
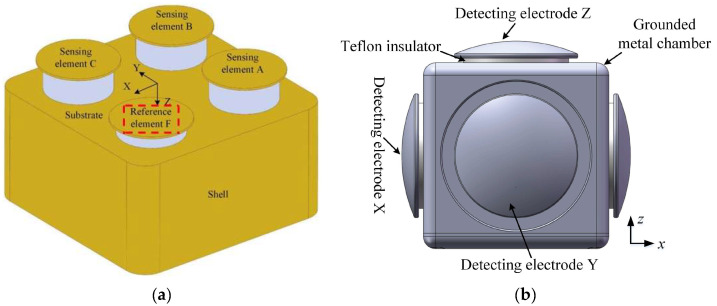
Two MEMS 3D EFSs in an assembled structure with (**a**,**b**) corresponding to [72,73], respectively.

In 2024, Zhang et al. designed a 3D EFS based on a coplanar structure that is resistant to charge interference [72], as shown in Figure 14a. This design aims to reduce the influence of space charge on electric field measurement and improve measurement accuracy. The relationship between the distance between the sealing cap and the sensing chip and the electric field was explored, and the influence of charge accumulation was overcome by calculating the difference between the output signals of the reference element and the sensing element. The maximum measurement error in the electric field range of 0–50 kV/m is 4.01%, and the linearity is better than 1%, demonstrating high measurement accuracy and stability. In 2024, Yang et al. developed a highly sensitive, moisture-proof 3D DC electric field microsensor [73], as shown in Figure 14b. This sensor adopts an inward-concave packaging structure and a unique moisture-proof design. The minimum sensitivity of the three measurement units of this EFS is 14.78 $mV\cdot kV^{-1}\cdot m$, which is 4.64 times higher than the highest sensitivity of previously reported MEMS 3D EFSs. Even under 99% RH conditions, the sensor can maintain linearity of less than 1%, and the maximum relative deviation is only 2.2% at any rotation posture. Compared to previous 3D EFSs, this sensor offers higher sensitivity, better humidity resistance, and the ability to maintain stable measurement accuracy in high humidity environments.

### 5.3. System-Level Integration and Application

The ultimate promise of MEMS 3D EFSs extends beyond fundamental device design and shrinking, encompassing their integration into functional systems for practical applications. Future development initiatives will concentrate on integrating these sensors into wearable or portable diagnostic and monitoring systems [74]. The intrinsic benefits of MEMS 3D EFSs—miniaturization, low power consumption, and scalability for mass production—render them exceptionally appropriate for system-level applications. Integrating 3D EFSs into portable health monitors facilitates electric field evaluations pertinent to personal health research [75]. In industrial settings, tiny 3D EFSs integrated into handheld devices can enable on-site EMC testing or safety inspections near high-voltage equipment [76]. The effective implementation of MEMS sensors in life-critical integrated systems [77] illustrates their commitment to downsizing and low power consumption, which nicely matches with the demands for practicality and ease of deployment in field applications of 3D EFSs. Systemic issues requiring resolution in these applications, such as dependable data collecting, wireless transmission, and power management, are critically pertinent to the future advancement of monitoring systems utilizing portable 3D EFSs.

## 6. Decoupling Calibration Method

A 3D EFS typically comprises a minimum of three autonomous sensing units, employed to quantify the electric field strength components along the *X*-, *Y*-, and *Z*-axes inside a rectangular coordinate framework. In practical applications, the output signal of a single sensing unit is influenced not only by the electric field component along the designated measurement axis but also by cross-interference from the electric field components along the other two non-corresponding axes. The response characteristics of the sensing unit measuring the electric field component along the *X*-axis are intricately linked to the electric field strength in the *X* direction and are also influenced by interference from the electric field components along the *Y*- and *Z*-axes. The inter-axis coupling effect presents a considerable obstacle to the precision of 3D electric field measurements. To effectively mitigate this issue and enhance the reliability of measurement data, two methods are typically employed to reduce the effects of inter-axis coupling interference: developing a decoupling matrix from an algorithmic standpoint or creating symmetry from a structural viewpoint.

### 6.1. Decoupled Calibration Matrix

In 3D EFSs, the sensing unit demonstrates linear properties in relation to each component of the applied electric field. This coupling characteristic can be formally expressed by the subsequent matrix equation, which precisely delineates the interaction among the components.

(8) $$\left[\begin{array}{c} V_{x}-V_{x_{0}}\\ V_{y}-V_{y_{0}}\\ V_{z}-V_{z_{0}} \end{array}\right]=\left[\begin{array}{ccc} k_{xx} & k_{xy} & k_{xz}\\ k_{yx} & k_{yy} & k_{yz}\\ k_{zx} & k_{zy} & k_{zz} \end{array}\right]\left[\begin{array}{c} E_{x}\\ E_{y}\\ E_{z} \end{array}\right]$$

The sensitivity matrix K is given as follows:

(9) $$K=\left[\begin{array}{ccc} k_{xx} & k_{xy} & k_{xz}\\ k_{yx} & k_{yy} & k_{yz}\\ k_{zx} & k_{zy} & k_{zz} \end{array}\right]$$

where $V_{i}$ is the output of the i-axis sensing unit, $V_{i_{0}}$ is the zero-point output of the i-axis sensing unit, and the sensitivity $k_{ij}$ represents the sensitivity of the electrostatic field in the j-direction on the i-axis sensing unit (where i=x,y,z and j=x,y,z). Therefore, the electric field E can be expressed as follows:

(10) $$\left[\begin{array}{c} E_{x}\\ E_{y}\\ E_{z} \end{array}\right]={\left[\begin{array}{ccc} k_{xx} & k_{xy} & k_{xz}\\ k_{yx} & k_{yy} & k_{yz}\\ k_{zx} & k_{zy} & k_{zz} \end{array}\right]}^{-1}\left[\begin{array}{c} V_{x}-V_{x_{0}}\\ V_{y}-V_{y_{0}}\\ V_{z}-V_{z_{0}} \end{array}\right]$$

The coupling matrix S is given as follows:

(11) $$S={\left[\begin{array}{ccc} k_{xx} & k_{xy} & k_{xz}\\ k_{yx} & k_{yy} & k_{yz}\\ k_{zx} & k_{zy} & k_{zz} \end{array}\right]}^{-1}$$

The measured electric field strength E is given as follows:

(12) $$E=\sqrt{E_{x}^{2}+E_{y}^{2}+E_{z}^{2}}$$

The electric field direction angle is calculated as follows:

(13) $$\phi =\cos^{-1}\left(\frac{E_{z}}{E}\right)$$

(14) $$\theta =\tan^{-1}\left(\frac{E_{y}}{E_{x}}\right)$$

where ϕ is the angle between the *Z*-axis and E, and θ is the angle between the horizontal projection of E and the *X*-axis.

### 6.2. Calibration Method

**Figure 15:**
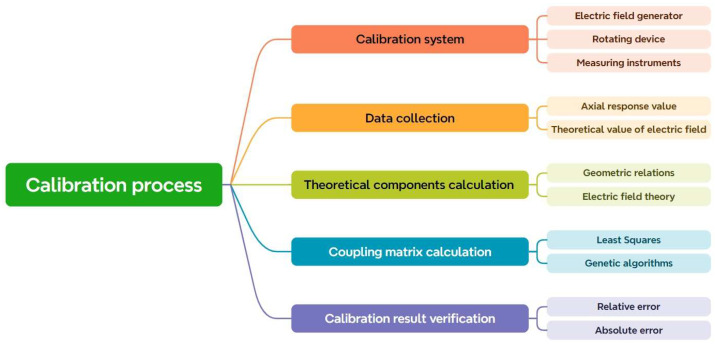
Coupling matrix calibration flowchart.

The sensitivity matrix K of the EFS can be derived via experimental calibration. The calibration procedure for the coupling matrix is depicted in Figure 15 and adheres to the following fundamental steps:

1. Calibration system construction: Construct a calibration system that can precisely regulate the direction and intensity of the electric field. This generally comprises a device that produces a uniform electric field and a rotating mechanism capable of firmly affixing and accurately rotating the sensor. The rotating apparatus often includes one or more orthogonal axes of rotation, enabling the sensor to be calibrated from various perspectives.
2. Data collection: Install the sensor within the calibration system and document the output data of the sensor under varying electric field orientations and intensities. It is imperative to maintain the consistency and stability of the electric field, together with the precise positioning and orientation of the sensor, during the data collection process.
3. Calculation of theoretical components: The theoretical components of the electric field in the sensor’s local coordinate system are computed based on the rotation angle and electric field strength, utilizing geometric relationships and electric field theory. These theoretical components will provide the foundation for comparison and calibration throughout the process.
4. Coupling matrix calculation: The gathered sensor output data and the computed theoretical electric field components are utilized to determine the coupling matrix via mathematical techniques, including the least squares method, genetic algorithm [78], and differential evolution algorithm [79]. The calculation of the coupling matrix constitutes an optimization problem aimed at identifying a matrix that minimizes the discrepancy between the sensor output, as transformed by the matrix, and the theoretical electric field components.
5. Calibration result verification: The coupling matrix derived from calibration is used to execute an inverse computation on the sensor’s output under alternative known conditions, thus acquiring the computed value of the electric field. The computed value is subsequently compared with the actual applied electric field value. The precision of the calibration outcome is assessed by computing the error (e.g., relative error, absolute error, etc.).

In calibration studies for 3D EFSs, it is crucial to consult international standards like IEEE 1309-2013 [80], which offer a systematic framework and methodological advice for sensor calibration. This standard delineates specific requirements for calibration systems, calibration techniques, and other essential components. Compliance with these criteria is essential for guaranteeing the traceability, comparability, and reliability of 3D EFS calibration outcomes, especially in contexts requiring high-precision measurements and data comparisons among various laboratories. Future 3D EFSs, particularly those aimed at industrial use, must ensure that the design and validation of calibration methods adhere comprehensively to the relevant standards’ guiding principles.

### 6.3. Calibration Applications and Limitations

Decoupling matrix calibration techniques are extensively employed in the calibration procedures of 3D EFSs [38,49,62,66,68,70,71,73]. This approach can efficiently mitigate inter-axis coupling interference; nonetheless, numerous obstacles and restrictions persist in real applications.

1. Environmental parameter drift: (a) Mechanical vibration interference: Motor vibrations induce irregular rotor motion, which disrupts the periodic shielding of the shielding structure and sensing plate, hence impacting the calibration of the decoupling matrix [38]. (b) Temperature drift: The Pockels effect in lithium niobate is influenced by temperature, although this is not dynamically corrected within the matrix, hence impacting calibration [49]. (c) Effects of humidity: Variations in environmental humidity induce drift in the dielectric constant, modifying capacitance values and leading to alterations in the sensitivity matrix. (d) Ground potential fluctuations: Conventional wired transmission induces variations in the ground potential of the signal conditioning circuit, hence impacting the precision of matrix calibration.
2. Calibration system errors: (a) Non-uniformity of the electric field: Edge effects in parallel plate calibration devices induce distortions in field strength, hence impacting matrix calibration.

## 7. Discussion

### 7.1. Current Advancements and Applications

Recent years have witnessed substantial advancements in the development of 3D EFSs, propelled by the want for accurate and extensive electric field measurements across diverse scientific and industrial applications. This research examines various sensors, including DC field mill, electro-optic effect, capacitive sensing, and MEMS 3D EFSs, each offering distinct benefits and serving significant roles in particular applications. DC field mill sensors have exceptional stability and resistance to interference, rendering them especially appropriate for prolonged monitoring in adverse conditions, such as high-voltage transmission lines [4,31,81,82,83,84,85]. They possess the ability to measure direct current electric fields. Nonetheless, their intricate design and necessity for mechanical rotation may restrict their applicability in specific contexts. Electro-optic effect sensors provide non-contact measurement capabilities [86]. Their robust resistance to electromagnetic interference renders them appropriate for detecting AC and transient electric fields in high-voltage settings [87]. The primary disadvantages include elevated expenses and considerable reliance on environmental factors, including temperature, humidity, and illumination. Capacitive sensing EFSs are adept at measuring swiftly varying electric fields, particularly those present in brief electromagnetic events. Nonetheless, in comparison to other sensor types, they have a restricted measuring range and diminished resilience to interference. MEMS sensors, characterized by their compactness, elevated sensitivity, and minimal power consumption, are ideally suited for compact wearable or embedded systems. Table 1 delineates the performance metrics and the advantages and disadvantages of 3D EFSs, categorized by their distinct operating principles and sensor architectures.

### 7.2. Challenges and Limitations

Despite significant progress, several challenges still remain in the development and application of 3D EFSs:

1. Sensitivity and resolution: Current 3D EFSs generally exhibit limited sensitivity and fail to satisfy the stringent detection demands for weak electric fields in domains such as biomedical imaging and nanoelectronics. Investigating novel sensing materials, refining sensor architectures, augmenting signal processing technologies, and further elevating sensitivity and resolution will be essential avenues for the future advancement of 3D EFSs.
2. Miniaturization and integration: Despite the advancements in MEMS technology facilitating the downsizing of 3D EFSs, numerous technical obstacles persist. The completed structure remains cumbersome due to the necessity of incorporating three electric field-sensitive chips, whereas single-chip packaging can modify the electric field distribution. Consequently, no viable solution has been established thus far. Attaining downsizing while maintaining sensor performance and reliability is a primary focus of contemporary research.
3. Inter-axis coupling interference: The output signal of each measurement axis in a 3D EFS is often affected by the electric field components from the other two orthogonal axes. This inter-axis coupling effect significantly impacts measurement accuracy. Although some studies have explored methods such as decoupling matrices and symmetrical structures combined with differential circuits to reduce coupling interference, these approaches still cannot fully eliminate the issue.
4. Space charge interference: In practical settings, space charge can result in charge accumulation on the sensor surface, hence impacting the precision of electric field measurements. While certain studies have suggested techniques, such as differential computations, to alleviate the effects of charge accumulation, investigations into the processes of space charge interference and related remedies are still scarce.

### 7.3. Future Research Directions

1. AI-augmented signal processing: Deep learning algorithms, including convolutional neural networks [88,89] and long short-term memory networks [90], can be developed to denoise, identify patterns, and extract features from raw electric field signals, thereby enhancing the detection sensitivity and precision of weak electric field signals. AI models can be trained to identify anomalous signals in real time by learning to detect nonlinear disturbances in electric fields within intricate surroundings.
2. Multi-source sensor fusion: The integration of 3D EFSs with environmental sensors, including temperature, humidity, and barometric pressure, alongside the application of fusion algorithms for the collaborative processing of multi-source data [91], effectively mitigates the interference caused by fluctuations in environmental parameters on electric field measurements. This enhances the system’s robustness and adaptability, making it particularly applicable for meteorological monitoring, disaster warning, and related domains.
3. Smart medical/environmental system-level integration applications: Three-dimensional EFSs can be integrated with wearable devices or implantable biosensors for the detection of human electrophysiological signals [92], such as cerebral or cardiac electric fields, thereby facilitating epilepsy prediction [93] and arrhythmia diagnosis. In environmental contexts, these sensors can be incorporated into edge AI devices [94] to enable intelligent monitoring and response to electromagnetic pollution, thunderstorm risks, and other environmental hazards.
4. 3D EFS productization/standardization: The productization and standardization of 3D EFSs necessitate the assistance of standardization efforts for their minimization and integration. This necessitates a robust emphasis on the authoritative function of international standards. Applicable standards, including IEEE 1309 [80], IEC 61786 [64], and the IEC 61000 series [95], offer essential rules and frameworks for the accurate calibration, performance assessment, electromagnetic compatibility validation, and final productization and standardization of sensors. Complying with and citing these standards is crucial for improving the environmental adaptability and market competitiveness of 3D EFSs.

## 8. Conclusions

This article provides a comprehensive review of 3D EFSs, encompassing DC field mill, electro-optic effect, capacitive sensing, and MEMS sensors. It encompasses their operational concepts, structural designs, decoupling calibration methods, and application examples. Three-dimensional EFSs are crucial in meteorology, power grids, and underwater detection; however, they encounter challenges such as inadequate sensitivity, complications in miniaturization and integration, inter-axis coupling interference, and space charge interference. In the future, innovations in related technologies are anticipated to enable 3D EFSs to make significant improvements in performance, cost efficiency, and adaptability. They are expected to advance in fields such as AI signal processing and sensor fusion, thereby broadening their application domains and enhancing support for scientific research and engineering practice.

## Figures and Tables

**Table 1:** Comparison of the performance of 3D EFSs with different structures and principles.

3D EFSs	Sensitivity/Cross-Axis Sensitivity	Measurement Range	Ref.	Advantages and Disadvantages
DC field mill	2 V/m	/	[32]	Advantages: High stability, strong anti-interference ability. Disadvantage: Narrow frequency band, mechanical wear, High power consumption.
	50 V/m	±30 kV/m	[35]	
	10 V/m	/	[37]	
Optical	$0.25\;mV/m-Hz^{1/2}$	/	[45]	Advantages: Non-contact measurement, strong anti-electromagnetic interference capability. Disadvantage: High cost, susceptible to temperature.
	X: 1.1 mV/kV/m Y: 1.7 mV/kV/m Z: 1.4 mV/kV/m	15–370 kV/m	[47]	
	$1\;\mu V\cdot {cm}^{-1}\cdot {Hz}^{-1/2}$	Maximum up to 6.5 MV/m	[50]	
	X: 0.908 mV/kV/m Y: 1.043 mV/kV/m Z: 0.781 mV/kV/m	X: 3.71 kV/m–388 kV/m Y: 2.78 kV/m–403 kV/m Z: 4.50 kV/m–375 kV/m	[51]	
	/	8–60 kV/m	[52]	
	0.0291 mV/(kV/m)	5 kV/m–50 kV/m	[53]	
Capacitive	3.03 mV/kV/m	<12 kV/m	[57]	Advantages: Broadband measurements, AC measurements. Disadvantage: Limited measuring range, weak anti-interference ability.
	19.10 mV/(kV · m^−1^)	1 kV/m–200 kV/m	[60]	
	0.1 mV/m 1 mV/m	5 mV/m–1 kV/m 500 mV/m–100 kV/m	/	
MEMS	$X:\;0.136\;mV\cdot kV^{-1}\cdot m$ $Y:\;0.121\;mV\cdot kV^{-1}\cdot m$ $Z:\;0.101\;mV\cdot kV^{-1}\cdot m$	0–50 kV/m	[66]	Advantages: Miniaturization, high sensitivity, low power consumption. Disadvantage: High manufacturing costs.
	X: 0.2315 mV/kV/m Y: 0.3727 mV/kV/m Z: 2.187 mV/kV/m	0–50 kV/m	[68]	
	5.48%	0–120 kV/m	[70]	
	19.54%	0–120 kV/m	[71]	
	14.78 mV · kV^−1^	/	[73]	
